# READY OR NOT: A DRAMA THERAPY STUDENT’S PERSPECTIVE ON WORKING WITH OLDER ADULTS

**DOI:** 10.1093/geroni/igad104.0491

**Published:** 2023-12-21

**Authors:** Camryn Hafner

**Affiliations:** New York University, New York City, New York, United States

## Abstract

Drama therapy has the potential to positively influence the lives of older adults in many facets, i.e., improving self-perceptions of aging (Dokter & Sajnani, 2023), attention, depressive symptoms, and overall quality of life (Lin et al., 2022). But drama therapists may also inadvertently cause harm, reenacting narratives of infantilization and indignity, or reinforcing the socially prescribed roles available to older adults (Dokter & Sajnani, 2023). Training and education may minimize harm and maximize positive outcomes; however, despite growing numbers of drama therapists and drama therapy interns working with older populations, the training of drama therapists minimally reflects this shift. Currently, a total of 800 internship hours, representing work with at least two distinct populations, are required to graduate from New York University with a Master of Arts in Drama Therapy and to qualify for a provisional license. While older adults may represent one of these populations, no courses in aging are required. One elective course, Drama Therapy and Aging: From Midlife to Elderhood, is offered once every two years. In a two-year program, this course may only become available after a student has begun their fieldwork. In this presenter’s case, the aging elective was taken concurrently with the start of her fieldwork at an assisted living facility working with persons living with dementia on a secured unit. Benefits and limitations of this student’s preparation to provide successful therapeutic experiences for individuals in this environment are considered, as well as the perceived implications for their future career as a drama therapist.

